# Validation of a competence-based assessment of medical students’ performance in the physician’s role

**DOI:** 10.1186/s12909-019-1919-x

**Published:** 2020-01-07

**Authors:** Sarah Prediger, Kristina Schick, Fabian Fincke, Sophie Fürstenberg, Viktor Oubaid, Martina Kadmon, Pascal O. Berberat, Sigrid Harendza

**Affiliations:** 1 III. Department of Internal Medicine, University Medical Center Hamburg-Eppendorf, Hamburg, Germany; 2 TUM Medical Education Center, School of Medicine, Technical University of Munich, Munich, Germany; 3 Department of Medical Education and Educational Research, Faculty of Medicine and Health Science, University of Oldenburg, Oldenburg, Germany; 4 German Aerospace Center (DLR), Hamburg, Germany; 5 Faculty of Medicine, University of Augsburg, Deanery, Augsburg, Germany

**Keywords:** Argument-based validation, Competences, Competence-based assessment, Performance-based assessment, Psychological assessment, Simulation

## Abstract

**Background:**

Assessing competence of advanced undergraduate medical students based on performance in the clinical context is the ultimate, yet challenging goal for medical educators to provide constructive alignment between undergraduate medical training and professional work of physicians. Therefore, we designed and validated a performance-based 360-degree assessment for competences of advanced undergraduate medical students.

**Methods:**

This study was conducted in three steps: 1) Ten facets of competence considered to be most important for beginning residents were determined by a ranking study with 102 internists and 100 surgeons. 2) Based on these facets of competence we developed a 360-degree assessment simulating a first day of residency. Advanced undergraduate medical students (year 5 and 6) participated in the physician’s role. Additionally knowledge was assessed by a multiple-choice test. The assessment was performed twice (t_1_ and t_2_) and included three phases: a consultation hour, a patient management phase, and a patient handover. Sixty-seven (t_1_) and eighty-nine (t_2_) undergraduate medical students participated. 3) The participants completed the Group Assessment of Performance (GAP)-test for flight school applicants to assess medical students‘ facets of competence in a non-medical context for validation purposes. We aimed to provide a validity argument for our newly designed assessment based on Messick’s six aspects of validation: (1) content validity, (2) substantive/cognitive validity, (3) structural validity, (4) generalizability, (5) external validity, and (6) consequential validity.

**Results:**

Our assessment proved to be well operationalised to enable undergraduate medical students to show their competences in performance on the higher levels of Bloom’s taxonomy. Its generalisability was underscored by its authenticity in respect of workplace reality and its underlying facets of competence relevant for beginning residents. The moderate concordance with facets of competence of the validated GAP-test provides arguments of convergent validity for our assessment. Since five aspects of Messick’s validation approach could be defended, our competence-based 360-degree assessment format shows good arguments for its validity.

**Conclusion:**

According to these validation arguments, our assessment instrument seems to be a good option to assess competence in advanced undergraduate medical students in a summative or formative way. Developments towards assessment of postgraduate medical trainees should be explored.

## Background

In medical education, performance has been evaluated traditionally by relying on the observation and judgement of teachers and medical experts. The evaluation of many aspects of clinical training requires demonstration and observation of skills and behaviour and cannot be assessed with written tests [1]. According to Flexner’s report more than a century ago, a written exam may “have some incidental value; it does not touch the heart of the matter” [2]. With learning approaches becoming more competence-based, tests are considered to be significant, when students are confronted with concrete cases and have to show their ability to collect relevant information and to suggest diagnoses [3]. Kane et al. [4] argue for performance-based assessment as an effective way to solve problems, which are associated with the use of objective tests. In the last decade, a focus in medical education was on the standardization of direct observation for assessing learners complementing multiple-choice testing [5]. Different methods to evaluate performance in the health care professions have been tested [6]. For the assessment of skills, the formats objective structured clinical examination (OSCE) [7], mini-clinical evaluations (Mini-CEX) [8] and direct observation of procedural skills (DOPS) [9] have been integrated in undergraduate medial education. The trend in medical education is directed towards competency-based approaches to monitor the progress of medical students [10]. Yet, competence modelling and measurements in higher education bear many challenges due to their multidimensionality and multi-causality of conditions and effects [11]. Since competences are abstract and not directly measurable, workplace-based assessments like Mini-CEX and DOPS ease the evaluation of candidates’ competences while observing their performance of professional activities. Although such assessment formats take place in the real work situation, they are lacking standardization and cannot be used with larger numbers of participants at the same time [12]. The simulation of a first working day in the clinical environment, during which students show their competences by performance, seems to be an adequate and valid format to test competences needed for a successful transition from undergraduate to postgraduate medical training. Such a performance-based assessment model was established in 2011 in the Netherlands and Germany [13].

The chain of inferences from observed performances to assessment decisions includes interpretative arguments [14, 15]. To validate these arguments, convincing support for these inferences and assumptions needs to be provided [4]. The aim of this study was to provide a validation argument for our newly designed assessment, simulating the first working day of a resident in a hospital. Messick [16] argues for a comprehensive theory of construct validity, which addresses score meaning and social values in test interpretation and test use. Even though Shepard [15] claimed that the complexity of Messick’s framework could be overwhelming in utilisation, we consider all of Messick’s proposed aspects of validity for a comprehensive and universal view on our assessment. Due to the high complexity of academically acquired competences, e.g. in medicine, and to the multidimensionality of our different assessment instruments, it is not sufficient to focus only on the statistical data of construct validities of particular instruments with convergent and discriminant aspects, without taking discussions of context issues into account. The pilot project of our assessment format was already discussed with Kane’s approach of validation [14] for the aspects “scoring“’, “generalization”, “extrapolation”, and “interpretation” and showed good arguments for validity [13]. We developed this assessment format further towards a 360-degree assessment of advanced undergraduate medical students’ competences based on a number of facets of competence needed for the first year of residency [17]. Therefore, we use Messick’s [16] construct framework of six distinguishable aspects of validation (1: content validity, 2: substantive/cognitive validity, 3: structural validity, 4: generalizability, 5: external validity, 6: consequential validity) for our validation argumentation.

## Method

### Study setting

The establishment of our 360-degree competence-based assessment, which we discuss based on Messick’s framework of validation, was based on three steps and developed over 3 years. In a first step, we conducted a ranking study of facets of competence needed by physicians to define the content, which should be evaluated in our assessment (Step 1). Afterwards we established the assessment and evaluated the data in two rounds to improve the assessment structure and rating instruments (Step 2). Additionally, the participants completed the Group Assessment of Performance (GAP)-test for flight school applicants in t_1_ one day after the 360-degree-assessment to assess medical students‘ facets of competence in a non-medical context to evaluate convergent validity (Step 3). The Ethics Committee of the Chamber of Physicians, Hamburg, confirmed the innocuousness of this study with consented, anonymized, and voluntary participation (PV3649). Written consent was obtained from all participants.

#### Ranking study (step 1)

To design the content of our assessment, we explored, which facets of competence were defined to be important for beginning residents. We performed a ranking study of 25 facets of competence relevant for physicians with 102 internists and 100 surgeons from three German universities with different undergraduate medical curricula [18]. The participating physicians were asked to rank the 25 facets of competence in an online questionnaire with respect to their relevance for beginning residents. The resulting competence facets on rank 1 to 10 became the basis of the design of our assessment.

#### Simulation-based assessment (step 2)

**Fig. 1:**
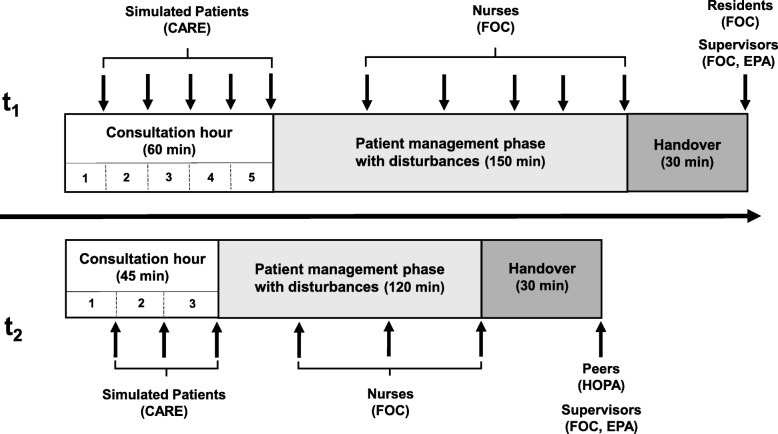
Phases of the competence-based 360-degree assessment and instruments. Note: CARE - Consultation and Relation Empathy, FOC - Facets of Competence, EPA - Entrustable Professional Activities, HOPA - Handover partner assessment; short arrows: moments of evaluation

The 360-degree assessment simulates the first working day of a resident [17] and was performed twice at the University Medical Center Hamburg-Eppendorf. In a first round (t_1_), 67 advanced undergraduate medical students (age: *M* = 26.05, *SD* = 2.18 years; 56.7% female) participated; of those, 26 students were at the end of their fifth year of a six-year undergraduate medical curriculum and 41 students were in their final (practice) year. In a second round (t_2_), the assessment took place with 89 medical students (age: *M* = 26.87, *SD* = 3.59 years; 67.4% female) in their final (practice) year. We recruited participants from three different German medical schools (Hamburg, Oldenburg, TU Munich). All students of the corresponding cohorts were invited by email and participants were assigned on a first come, first served basis. Their participation was voluntary and was rewarded with a book voucher of 25 €. Participants passed in this simulation through three phases, which were selected because of their typical characteristics of clinical routine: (1) a consultation hour with simulated patients during which their detailed histories were taken, followed by (2) a patient management phase, which included interactions with nurses and supervising doctors, and (3) a patient handover phase to a resident. The supervisors met their student in the role of a beginning resident three times: first, to welcome them before the consultation hour, second, in a short face-to-face interaction during the patient management phase, and third, during the patient handover in the role of a passive observer [13, 17]. During the patient management phase, the participants collaborated interactively with the nurses in typical clinical routine situations, e.g. interprofessional discussions (face-to-face or by telephone) about the patients seen by the participants during the consultation hour and one new patient. They could call their supervisor as well as the nurses to ask for support during phases 1 and 2 of the simulation. Finally, the participants handed over their patients to a real resident in the third simulation phase. Afterwards, debriefing rounds were performed with each participant group (t_1_: five participants, t_2_: six participants) to evaluate the assessment from the participants’ perspectives. Fig. 1 shows the three phases of assessment for t_1_ and t_2_. Arrows indicate the time points of evaluation by the different raters indicating the respective scoring forms.

Based on the analysis of data from t_1_, several changes were made for the assessment phase at t_2._ Since we found no significant differences in rating of competences between supervisors and residents at t_1_ [19], residents were no longer included as raters in the assessment and the handover was changed to a peer handover between participants, who had worked with different simulated patients. This change had no organizational reason, but rather resolved potential influences by residents, who partly interfered in the handover reports in t_1_, thus enabling participants to demonstrate clinical reasoning abilities. To implement this change to the handover phase at t_2_, we had to introduce two groups (A and B) of participants, who worked simultaneously with different patient cases in the consultation hour_._ During the handover, participants of group A handed over their patients to group B and vice versa. As a result, we reduced the number of simulated patients for the consultation hour from five to three, decreasing the consultation phase from 60 to 45 min. The time for the patient management was reduced from 2.5 to 2 h including a reduction of the number of disturbances from five to three, accordingly.

#### Group Assessment of Performance (GAP)-test (step 3)

The participants completed the Group Assessment of Performance (GAP)-test for flight school applicants at the German Aerospace Center (DLR) in Hamburg to assess medical students‘ facets of competence in a non-medical context [20]. GAP is a computerized problem solving simulation, during which four candidates work on a scheduling task. The participants were observed and assessed with a set of empirically derived behaviour checklists including teamwork, communication, and leadership by two experienced DLR aviation psychologists (more than 2000 prior assessments) who passed a one-day standardization seminar prior to this assessment [21]. Some facets of competence in the GAP-test are similar to our relevant facets of competence needed in clinical environment.

### Rating instruments

To evaluate the ten selected facets of competence, four main instruments were used: (1) a scoring sheet to assess facets of competence (FOC), used by supervisors, nurses, and residents, (2) a scoring sheet to assess entrustable professional activities (EPA), used by supervisors, (3) the Consultation and Relational Empathy questionnaire (CARE) [22] used by simulated patients, and (4) a questionnaire for a handover partner assessment (HOPA), used by peers. Table 1 shows, which facet of competence the respective instruments assessed.

**Table 1:** Overview of facets of competence assessed with the main instruments

Facets of competence	Instruments	
	direct	indirect
Responsibility The physician takes responsibility and shows accountability for his work. He/She accepts liability for his/her work.	FOC	EPA
Teamwork and collegiality The physician cooperates effectively and respectfully in a (multidisciplinary) team, taking the views, knowledge, and expertise of others into account.	FOC	EPA, HOPA
Knowing and maintaining own personal bounds and possibilities The physician knows the boundaries of his own ability and asks for help (timely) when needed. He/She reflects on himself/herself and the situation.	FOC	EPA, HOPA
Empathy and openness The physician shows empathy, openness and susceptibility/accessibility in his/her contact with patients.		EPA, CARE
Structure, work planning and priorities The physician sees the overall picture, has organizational skills and a flexible attitude, and sets priorities in his/her work.	FOC	EPA, HOPA
Coping with mistakes The physician is aware of the fact that anyone can make and does make mistakes once in a while. He/She is approachable when someone points out his/her mistakes and reacts adequately when he/she thinks that a colleague makes a mistake.	FOC	EPA, HOPA
Active listening to patients The physician listens actively to patients and reacts (verbally and nonverbally) on the things he/she hears in a way that encourages the sharing of information (by the patients) and confirm his/her involvement with the patient. He/She shows attention to non-verbal signals coming from the patients.	CARE	EPA
Scientifically and empirically grounded method of working The physician uses evidence-based procedures whenever possible and relies on scientific knowledge. He/She searches actively and purposefully for evidence and consults high-quality resources. He/She uses his scientific knowledge critically and carefully in his/her work.	FOC	EPA
Ethical awareness The physician is acquainted with ethical aspects of his/her work. He/She distinguishes different points of view in the moral debate and makes deliberate choices when his/her work confronts him/her with ethical issues		EPA
Verbal communication with colleagues and supervisors The physician gives structured, pithy, and unambiguous verbal reports on his/her findings on a patient and his diagnostic and therapeutic policy. He/She asks relevant and purposeful questions.	FOC	EPA, HOPA

Direct: facet of competence is explicitly assessed (FOC, CARE); indirect: facet of competence is implicitly assessed by patient case vignettes (EPA) or anchor examples (HOPA, CARE)

FOC scoring sheets directly assess facets of competence by observing performance during phases 2 and/or 3 with 5-point scales from 1 “insufficient” to 5 “very good”. Besides rating the facets of competence, supervisors and nurses had to evaluate the confidence of their judgement for every facet of competence on the FOC-scoring sheets.

Additionally, participants’ performance was the basis for indirect assessment by the supervisors using the following EPA scoring form: twelve small case vignettes are described and the supervisor rater had to indicate the level of entrustment for each participant and case (1: no permission to act, 2: permission to act with direct supervision (supervisor present in the room), 3: permission to act with indirect supervision (supervisor not present in the room, but quickly available if needed), 4: permission to act under distant supervision (supervisor not directly available, but a telephone call is possible, i.e. “unsupervised”), 5: permission to provide supervision to junior trainees) [23].

The HOPA questionnaire consists of items evaluating several facets of competence and items evaluating aspects of clinical reasoning with 5-point scales from 1 “insufficient” to 5 “very good”. Additionally, participants were asked if they had known their handover-partner before the assessment day, which was hardly the case.

Clinical reasoning, the cognitive process of getting to the solution of a patient case, was evaluated with the validated post-encounter form (PEF) [24]. One PEF was used by the participants per patient case and the forms were filled out during the patient management phase of the assessment.

To measure medical knowledge, the participants completed a multiple-choice test with 100 case-based questions with one correct answer out of five answers per question. The 100-item knowledge test was compiled from 1000 freely available United States Medical Licensing Examination Step 2 type questions including case vignettes [25].

### Procedure of rating

Simulated patients, nurses, supervisors, and residents or peers, respectively, assessed facets of competence of advanced undergraduate medical students in the role of beginning residents based on interaction or observation using several instruments. We trained all raters for using the respective instruments with a standardised rater training. This training included practice with all rating instrument including the assessment of roleplays or videotaped physician-patient interaction situations with competent and less competent performances and the discussion of assessment judgements to substantiate a standardised rating. Patient cases and case vignettes for EPA assessment were constructed by adapting real patient cases to the assessment setting [17]. They were discussed in detail during the supervisor rater training. Each simulated patient filled out the CARE questionnaire directly after every individual consultation (t_1_: five questionnaires per participant, t_2_: three questionnaires per participant). Nurses filled out FOC scoring sheets for each disturbance (t_1_: four per participant, t_2_: two per participant) and for a total rating per participant at the end of the patient management phase. Supervisors completed FOC scoring sheets for every participant per patient (t_1_: five, t_2_: three) and for a total rating after the handover. The interrater reliability for the pilot FOC scoring, where two supervisors assessed the same participant, had been excellent [13] allowing for rating with one rater per assessor group in our setting. Residents used one FOC scoring sheet only for overall rating after the handover. Finally, supervisors completed the EPA form after they had seen the participants off. In t_2_, peers filled out HOPA scoring sheets after the handover.

### Analysis of validity

Following Messick’s argument-based approach of validation [16], we examined structural validity, parts of cognitive validity, and generalizability by discussing our established assessment structures in comparison to the underlying theoretical assumptions. Additionally, statistical analyses for content validity, convergent validity, and other parts of cognitive validity were conducted with SPSS Statistics 23. We do not provide arguments for consequential validity because of its prognostic value, which can only be assessed through longitudinal observation of participants. Aspects of content validity were analysed by a comparison of our ranking study of facets of competence with respect to their relevance for beginning residents [18] with an earlier Delphi study [13]. To examine parts of cognitive validity, we analysed differences between the assessment of confidence of judgment between t_1_ and t_2_ by conducting a t-test as well as effect sizes (Cohen’s *d*) for both rating groups. To analyse differences between the FOC-assessment of supervisors, nurses, and peers in t_2_, we conducted an analysis of variance (ANOVA) and a Bonferroni post-hoc test. Cronbach’s *α* was calculated for reliability of FOC-assessment scores (t_1_ and t_2_) and HOPA-assessment scores (t_2_). To verify convergent validation, Pearson’s correlation coefficient (*r*) between the assessed competences of 360-degree assessment respectively EPA (t_1_) and GAP-Test were computed.

## Results

### Evidence for structural validity

**Fig. 2:**
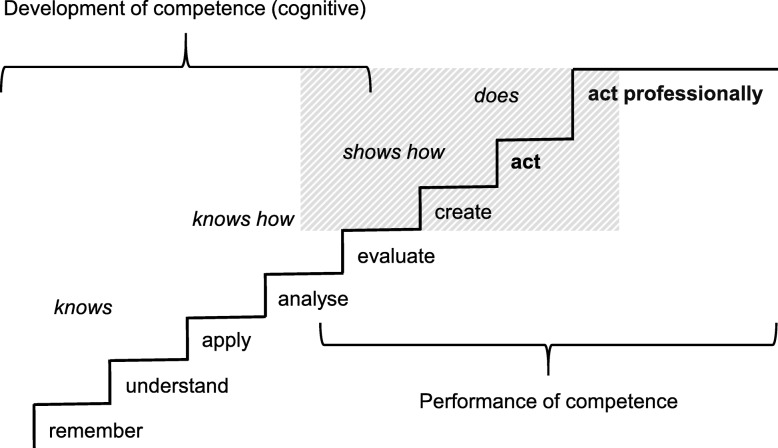
Constructs of competence for our 360-degree competence-based assessment. Note: italic above the staircase: steps from Miller’s framework for clinical assessment [28]; below the staircase: steps from Bloom’s taxonomy [27]; bold below the staircase: additional steps, shaded area: the setting of our 360-degree assessment

According to the argument-based approach of validation, we discuss the theoretical construct, in alignment with its realization in our assessment setting. Our 360-degree assessment is built on a theoretical construct of Bloom’s taxonomy [26, 27] combined with Miller’s framework for clinical assessment [28]. It can be categorized between Miller’s categories “shows how” and “does” (Fig. 2).

The assessment, resembling a clinical workplace, tests more than just skills like an OSCE (level “shows how” or “analyse” and “evaluate” according to Bloom). At the same time, it does not completely cover Miller’s level “does” in terms of assessing a candidate in the real work place. Yet, our 360-degree assessment model is operationalised as a realistic simulation of a resident’s working day, with real patient cases, performed by professional actors. The candidates’ performance includes Bloom’s level “create” and one additional level “act”, which comprises the relevant knowledge and skill without the necessity to assess them separately. For the competence levels of “shows how” and “does”, participants (in the role of residents) need to master the basic steps of cognitive competence development. They also need to be able to perform clinical reasoning, which is the typical cognitive process for solving patient cases based on information gathered by history taking, physical examination, and other investigations [29] - like they do in our assessment.

Miller’s “knows” and Bloom’s “remember” as well as “understand” are depicted in the multiple-choice knowledge test. Miller’s “knows how”, Bloom’s “apply” and “analyse”, are assessed by the CARE-questionnaire. In our simulation, Miller’s “shows how” and parts of the “does” level are covered when participants “evaluate“ patient cases further during the management phase, e.g. by ordering blood tests, and “create“ treatment suggestions, which are justified by clinical reasoning as measured with the validated post-encounter form (PEF) [24]. Participants also had to “act” in making telephone calls or dealing with interprofessional requests and they actually felt responsible for the wellbeing of the patients, as they stated in the debriefing rounds. This indicates that our assessment is operationalised close to Miller’s “does”-level. Responsibility and other facets of competence are necessary for the participants to act professionally. They need to perform well in those facets of competence required by beginning residents in order to handle the tasks they will be entrusted with. The facets of competence mostly correlate significantly with each other, which indicates associated underlying constructs and within-item dimensionality [30]. The facets of competence assessed directly with FOC scoring sheets by observing performance, require Bloom’s “analyse”, “evaluate” and “create”. Additionally, the observed performance is the basis for indirect assessment of entrustable professional activities (EPA). While competences refer to abilities, EPAs are “units of professional practice” [23], which integrate several competences and consist of different aspects of knowledge, skills and attitudes [31]. In summary, our instruments measuring FOCs and EPAs include different aspects of competence, which become observable on a high level with respect to Miller’s and Bloom’s taxonomies in the assessment performance.

### Evidence for content validity

To examine content validity and explore, which facets of competence are *sustainably* identified as being important for beginning residents, we compared the results of our ranking study [18] with an earlier international study, which included medical educators from Germany and the Netherlands [32]. The ten competences defined as the most relevant ones for beginning residents were similar in both studies (Table 2). Only “Structure, work planning and priorities” and “Ethical awareness” were ranked higher in 2017 compared to 2013 (rank 5 versus rank 16 and rank 9 versus rank 17, respectively). In the previous study, assessments by medical educators from the Netherlands and Germany were combined for the final ranking [32]. Considering German educators’ assessment alone, the competence “Structure, work planning and priorities” was already ranked among the top ten facets of competence in the previous study (i.e. rank 8) [32]. With an agreement on eight, respectively nine out of ten facets of competence important for beginning residents [18], the content validity is very high for our 360-degree assessment model. The increasing awareness among physicians of rising economic pressure leading to a deterioration in patient-orientation [33] may have led to a higher ranking of ethical awareness compared to the previous study. In summary, the underlying facets of competence seem to represent our construct of medical competence needed for the first year of residency adequately.

**Table 2:** Comparison of ranking orders of the ten main competences

Competences	2017^a^	2013^b^
Responsibility	1	8
Knowing and maintaining own personal bounds and possibilities	2	2
Teamwork and collegiality	3	4
Empathy and openness	4	7
Structure, work planning and priorities	5	**16**
Coping with mistakes	6	9
Active listening to patients	7	5
Scientifically and empirically grounded method of working	8	1
Ethical awareness	9	**17**
Verbal communication with colleagues and supervisors	10	6
Safety and risk management	**12**	10
Active professional development	**13**	3

^a^ [18], ^b^ [32]

bold = rank higher than 10

### Evidence for cognitive validity

#### Participants’ cognition

For cognitive validity, which complements content validity, not only the content of an assessment has to be adequately represented, but the approach towards solutions to questions or problems have to be equally considered [34]. Therefore, it is important to achieve a good fit between the theoretical construct and the actual cognition of an assessment. To enable participants to show their facets of competence, our assessment had to be operationalized within a typical medical setting and resembles a first working day of a new resident with all relevant processes a beginning resident would encounter (history taking, patient management, and handover). Additionally, the typical disturbances in clinical daily routine, i.e. interprofessional interactions, telephone calls etc., were included to make the setting even more realistic. The patient cases were constructed in such a way, that pattern recognition followed by analytical thinking was necessary in the clinical reasoning process. Clinical reasoning, the typical cognitive process to get to the solution of patient cases, is based on information from history taking, physical examination, and other investigations [29]. Each of these steps requires a combination of different facets of competence. To show clinical reasoning abilities, it is not necessary to entirely solve a patient case but to provide comprehensible reasons for the different steps of work-up during the patient management phase. Additionally, comprehensible reasons for a patient’s further work-up or treatment can be observed during the handover. Competent behaviour cannot be displayed without specific knowledge. A regression analysis with data from our 360-degree assessment showed that the medical knowledge of our participants, represented by their results in the multiple-choice test, questions, explained 11% of the variance of clinical reasoning skills [35].

#### Assessors’ cognition

The possible cognitive influences of the assessors’ perspective need to be considered in the context of the cognitive aspects of assessment’s validity. The rating basis for the main assessment instruments was the observation by different rating groups, who were also interactively involved in the simulation model. In addition to first impressions [1] and rating context [36], individual mental models of performance assessment especially influence rater-based assessment [37]. To build shared mental models, all assessors discussed the facets of competence during rating trainings. Internal consistency of the total FOC score over all assessors was satisfying for each rating group in t_1_ (Cronbach’s *α*: supervisors = .90, residents = .80, nurses = .78) [19], and there are hardly any significant differences of the means in FOC sores between t_1_ and t_2_. During t_1_, the facets of competence “Coping with mistakes” and “Scientifically and empirically grounded method of working”, were most frequently marked with “judgement not possible” [19]. This could result from their arguable meaning or from lack of possibilities to observe these facets of competence in participants. To reduce raters workload and to support rating validity [38], we described examples of observable behaviour as anchors for the seven facets of competence in addition to the definitions for each item, and complemented them as a second sheet to the FOC scoring forms for the assessment at t_2_ and in the rater training. The comparison of judgement confidence between t_1_ (assessment without) and t_2_ (assessment with additional anchors) showed that supervisors felt more confident at t_2_ in all FOC assessments (significantly in four out of seven) and nurses in four FOC assessment decisions (significantly in two out of six) (Table 3). Providing additional anchors seems to have improved assessors’ work with the FOC scoring form. Especially the rating of “Responsibility” was eased for supervisors’ assessment and showed 16.8% less ratings of “judgement not possible” (Table 4). On the other hand, the facets of competence “Coping with mistakes” and “Scientifically and empirically grounded method of working” were even more frequently marked as “judgement not possible” (supervisors + 6.8% and + 39.4%, respectively, nurses: 30.4%) at t_2_ than at t_1_. Internal consistency of the FOC total scoring over all assessors per rating group was satisfying with a Cronbach’s *α* at t_2_ with all facets of competence for supervisors (.94) and without “Coping with mistakes” for nurses (.76). This weakness in Cronbach’s *α* for the assessor group of nurses might have occurred because “Coping with mistakes” was assessed less frequently by them at t_2_. This leads us to the conclusion that the assessment instrument works well with the new anchors and some aspects of the assessment will need to be adapted to make two facets of competence “coping with mistakes” and “scientifically and empirically grounded method of working” more observable.

**Table 3:** Comparisons of confidence of judgement

Facets of Competences		Supervisors				Nurses			
	t	*M* ± *SD*	*p*^a^	*d*_Cohen_	*N*	*M* ± *SD*	*p*^a^	*d*_Cohen_	*N*
Responsibility	1	3.47 ± 0.79	.605	.086	55	4.23 ± 0.76	.121	.254	66
	2	3.56 ± 1.19			84	4.42 ± 0.74			86
Teamwork and collegiality	1	3.04 ± 1.21	.001	.604	57	4.25 ± 0.75	.799	.043	65
	2	3.73 ± 1.09			78	4.22 ± 0.64			83
Knowing and maintaining own personal bounds and possibilities	1	3.64 ± 0.90	.019	.389	67	3.74 ± 1.02	.303	.174	65
	2	3.99 ± 0.90			87	3.91 ± 0.94			69
Structure, work planning and priorities	1	4.04 ± 0.59	.266	.196	67	3.76 ± 0.95	.047	.334	67
	2	4.16 ± 0.63			90	4.06 ± 0.85			80
Coping with mistakes	1	2.77 ± 1.25	.034	.400	52	4.16 ± 0.81	.005	.599	43
	2	3.31 ± 1.43			61	4.62 ± 0.73			50
Scientifically and empirically grounded method of working	1	2.47 ± 1.03	<.001	1.246	51	4.23 ± 1.11			39
	2	3.90 ± 1.26			48	–			–
Verbal communication with colleagues and supervisors	1	4.09 ± 0.67	.634	.082	67	4.31 ± 0.70	.144	.243	67
	2	4.15 ± 0.78			89	4.14 ± 0.70			83

Note: Based on analysis of t_1_ data, we noticed, that nurses had no possibility to assess “Scientifically and empirically grounded method of working” in our simulation. Therefore, this competence was deleted from the scoring sheet in t_2_

*p*^a^: Significances for the differences between t_1_ and t_2_

**Table 4:** Frequencies of rating decisions with judgement not possible

	Judgement not possible									
	Supervisors					Nurses				
	t_1_		t_2_		Δ (%)	t_1_		t_2_		Δ (%)
Facets of competences	*N*	%	*N*	%		*N*	%	*N*	%	
Responsibility	12	17.9	1	1.1	-16.8	0	0	3	3.3	3.3
Teamwork and collegiality	10	14.9	11	12.2	-2.7	0	0	1	1.1	1.1
Knowing and maintaining own personal bounds and possibilities	0	0	2	2.2	2.2	4	6.0	17	18.9	12.9
Structure, work planning and priorities	0	0	0	0	0	0	0	6	6.7	6.7
Coping with mistakes	17	25.4	29	32.2	6.8	31	46.3	69	76.7	30.4
Scientifically and empirically grounded method of working	19	28.4	61	67.8	39.4	45	67.2	–	–	–
Verbal communication with colleagues and supervisors	0	0	0	0	0	0	0	3	3.3	3.3

For the HOPA, the peer assessment instrument used after the handover at t_2_, a Cronbach’s *α* of .73 showed acceptable internal consistency. However, peers assessed several facets of competence significantly better than nurses and supervisors with the FOC. This supports the finding that peer-assessment cannot replace teacher-assessment in high-stake decisions about students [39] but has its place in formative assessment when peers act as tutors in certain medical learning environments [40].

### Evidence for generalizability

As described in detail above, our assessment model is designed as an authentic simulation of a resident’s first working day with all relevant phases (history taking, patient management, and handover) except for physical examination. Therefore, it is highly representative for real work in a hospital and generalizable, even though it has to be considered that no complete standardization could be achieved because of the ever-changing, unpredictable clinical context [32]. However, participants are faced with different patients and tasks of the daily clinical routine, providing the possibility to show different competences required in different situations, which reduces variance caused by task specificities. Additionally, the same professional actors, trained as standardized patients, played the patient cases for all participants. Furthermore, the simulation is independent of assessors (section Evidence for cognitive validity) and participants. This provides the option to assess advanced undergraduate medical students but also residents at different stages of training with our 360-degree assessment tool.

### Evidence for external validity

As one external aspect of validity, we focus on convergent validity as part of construct validity. The students, who participated in our assessment at t_1_, also passed the validated Group Assessment of Performance (GAP)-test at the German Aerospace Center (DLR) in Hamburg. Facets of competence measured in our 360-degree assessment correlate with competences assessed with GAP (Table 5). The moderate correlation between “Verbal communication with colleagues and supervisors” and GAP’s “Communication”-item suggests similarities in operationalisation and validation of this facet of competence. The items measuring “Teamwork” in the two assessments do not correlate significantly, hence, different underlying conceptualisations can be assumed. In the GAP-test, observing raters assessed participants interacting with team partners. In our 360-degree assessment, raters were part of the simulation and evaluated the teamwork they experienced. The different perspectives might have led to different ways of evaluation.

**Table 5:** Correlations between facets of competence of 360-degree assessment (ÄKHOM) and GAP

Facets of competence (ÄKHOM)	Facets of competence (GAP)					
	Teamwork		Communication		Leadership	
	*r*	*p*	*r*	*p*	*r*	*p*
Responsibility						
Supervisors	.038	.791	.261	.064	.176	.217
Residents	.097	.497	**.337**	**.016**	**.383**	**.006**
Nurses	.181	.153	.136	.282	.239	.057
Teamwork and collegiality						
Supervisors	-.011	.937	.166	.232	.113	.418
Residents	-.045	.738	.145	.277	.116	.386
Nurses	.204	.106	.291	.019	.240	.056
Verbal communication with colleagues and supervisors						
Supervisors	.140	.271	**.261**	**.037**	.221	.079
Residents	**.266**	**.035**	**.321**	**.010**	**.254**	**.045**
Nurses	.060	.639	.229	.069	**.275**	**.028**

bold = significant differences

“Responsibility” in the 360-degrees assessment and “Leadership” in the GAP-test show a significant correlation, indicating similar conceptualisation, since responsibility is an essential part of (clinical) leadership [41]. Furthermore, “Leadership” from the GAP-test correlates moderately with “Verbal communication with colleagues and supervisors” from the 360-degree assessment. As operationalised for the EPA-questionnaire, observation of responsibility in a participant is highly relevant for the level of entrustment given to a participant for a specific EPA by an assessor and “Leadership” shows significant moderate correlations with six of the 12 assessed EPAs (Table 6). Additionally, four EPAs correlate significantly with “Communication”. In summary, we provided arguments for convergent validity, especially for “Communication” and “Responsibility”/“Leadership”.

**Table 6:** Correlations between EPA of 360-degree assessment (ÄKHOM) and GAP

	Facets of competence (GAP)					
	Teamwork		Communication		Leadership	
EPA	*r*	*p*	*r*	*p*	*r*	*p*
Emergency treatment of acute cardiac insufficiency	.181	.152	.085	.506	.194	.124
Handling a patient’s complaint	.094	.460	.070	.582	.213	.091
Pre-operative information and consent	.088	.490	**.425**	**<.001**	**.343**	**.006**
Breaking bad news	.133	.295	.177	.163	**.357**	**.004**
Clinical decision making on acute infection	-.058	.649	.175	.167	.096	.405
Solving a management problem	-.007	.956	.164	.195	**.316**	**.011**
Acting on suspicion of self-indicated illness	.032	.799	**.246**	**.050**	**.248**	**.048**
Treatment of a critically ill patient	-.154	.223	.041	.746	-.042	.740
Interaction with a consultant	.108	.395	**.550**	**.042**	.198	.116
Presentation of an oncology patient at a tumour board	.145	.254	**.309**	**.013**	**.326**	**.009**
Medication error	.043	.737	.113	.373	.172	.175
Acting on patient’s will	.045	.724	.229	.069	**.309**	**.013**

bold = significant differences

## Discussion

The pilot project of our assessment format was already discussed with Kane’s approach of validation [14] and showed good arguments for validity [13]. We re-designed the assessment to become a full 360-degree assessment and used Messick’s construct framework of six distinguishable aspects of validation [16] to address central issues of the concept of validation of the underlying competences assessed. Content, cognitive, structural and external aspects of validity as well as generalizability were evaluated and found to be sufficiently represented in our assessment. Only the prognostic aspect of consequential validity, Messick’s sixth aspect of validity [16, 34], could not completely be answered with our competence measurement concept. Consequential predictions from assessments are the most difficult part of empirical validation, because career success can be defined in various ways and is characterized by objective/extrinsic and subjective/intrinsic career success [42]. Additionally, assessment of career success needs a longitudinal approach and is difficult to predict with a single simulation.

However, we found arguments for a validity for the other five aspects of Messick’s approach. The comparison of assessment results with those from a validated instrument, the Group Assessment of Performance (GAP)-test [20], to demonstrate convergent validity showed moderate concordance in competence assessment scores. Even though the performance of competence was different in the two assessments, group work and passive observation during the GAP-test and interactive individual work and involved assessors during our 360-degree simulation assessment, the corresponding results provide an argument for convergent validity of our assessment, even though we could only show moderate correlation. According to Messick [16], evidence for structural validity can be provided for our simulation assessment model, which has been demonstrated to be well operationalised to enable participants to perform on the higher levels of Bloom’s taxonomy [26, 27] and Miller’s framework for clinical assessment [28]. Solid content validity is provided for our assessment, since the ten facets of competence, on which our assessment instruments are based, were consistently rated as being important for beginning residents throughout the past 6 years [18, 32]. Nevertheless, if the relevance of facets of competence for beginning residents changed over time, our assessment could be easily adjusted for new aspects in patient cases or management tasks. For instance, the ‘newly’ included facet of competence “Ethical awareness” [18], which was not part of the pilot project [13] is currently only indirectly assessed with the EPA-instrument. To avoid this limitation, an adaptation of the ethical awareness scale for nurses [43] could be included in our assessment in combination with adapted management tasks to directly observe ethical awareness in our assessment. The participants’ cognitive process of clinical reasoning (Messick’s sustainable aspect of validity) is facilitated by our assessment structure independent of the content of the patient cases and their degree of difficulty. Good clinical reasoning, evaluated with post-encounter forms (PEF) in our assessment, correlates positively with knowledge and teamwork [35]. Since written handover can improve the clinical reasoning process and increase the accuracy of information transfer [44], the PEF appears to be a very useful instrument to validate the assessment of cognitive processes. The possibility to demonstrate and to assess clinical reasoning skills was improved at t_2_ by changing the handover setting to a peer handover between participants. This followed the demand to improve educational interventions to test areas of competence (i.e. clinical reasoning), where medical students have been found to be ill prepared [45]. Therefore, good cognitive aspects of validity seem to be present in our assessment structures. Additionally, the structural changes made between t_1_ and t_2_ (reducing the number of patients and the number of interprofessional interactions per participant and shortening the time of the management phase) seem to have had no negative impact on the quality of the assessment (i.e. the cognitive validity).

A limitation of our assessment in simulating the working day of a real resident is that it lacks physical examination of the simulated patients. The results of the respective physical examination are provided in written form, instead. The skill to elicit the correct physical findings of a patient is important in combination with history taking to start the clinical reasoning process. Many universities already assess physical examination skills in OSCEs [46, 47]. Hence, our competence-based assessment does not necessarily have to test this skill. At the same time, different results are achieved for the same physical examination skill when assessed at different universities [48]. Therefore, providing physical examination results in writing created equal conditions for all participants from the three different medical schools in our assessment. Another weakness of our study is the use of the PEF which was validated for second year students [24]. However, it was already successfully used for final year medical students in a previous study [13]. A strength of our 360-degree assessment is that it is based on internationally acknowledged facets of competence relevant for beginning residents [18, 32]. It could also be adequately used as complementary formative assessment during undergraduate medical education. Our participants expressed a strong interest in receiving feedback with respect to their performance to be able to improve certain facets of competence during their further studies. This provides an additional generalizability argument to use our 360-degree assessment in undergraduate or postgraduate medical education.

## Conclusions

We could provide arguments for most of Messick’s aspects of validity for our newly designed 360-degree competence-based assessment for undergraduate medical students. This simulation and its assessment instruments can be used to evaluate ‘medical competence’ in advanced undergraduate medical students in a summative or formative way. Since the validity of this assessment was independent of the content and the difficulty of the patient cases and management tasks, its further development for use during postgraduate medical education and the assessment of residents should be explored.

## Data Availability

All data and material are available from the manuscript, from published studies from the ÄKHOM project cited in the references or from the corresponding author upon request.

## Abbreviations

ÄKHOM: Ärztliche Kompetenzen: Hamburg, Oldenburg, München (Medical Competences: Hamburg, Oldenburg, Munich)
CARE: Consultation and Relational Empathy
DOPS: Direct observation of procedural skills
EPA: Entrustable Professional Activity
FOC: Facets of Competence
GAP-test: Group Assessment of Performance test
HOPA: Handover Partner Assessment
Mini-CEX: Mini-clinical evaluations
OSCE: Objective structured clinical examination
PEF: Post-Encounter Form
